# Inactivation of MS-2 virus in water by rotational generator of hydraulic shock

**DOI:** 10.1016/j.heliyon.2024.e39795

**Published:** 2024-10-24

**Authors:** Benjamin Bizjan, Gašper Rak, Sabina Kolbl Repinc, Polonca Ropret, Janez Kosel

**Affiliations:** a University of Ljubljana, Faculty of Mechanical Engineering, Aškerčeva 6, 1000, Ljubljana, Slovenia; b University of Ljubljana, Faculty of Civil and Geodetic Engineering, Jamova 2, 1000, Ljubljana, Slovenia; c National Institute of Chemistry, Hajdrihova 19, 1000, Ljubljana, Slovenia; d Institute for the Protection of Cultural Heritage of Slovenia, Poljanska 40, 1000, Ljubljana, Slovenia; e Smithsonian Museum Conservation Institute, 4210 Silver Hill Road, Suitland, MD, 20746, USA; f University of Ljubljana, Faculty of Chemistry and Chemical Technology, Večna pot 113, 1000, Ljubljana, Slovenia

**Keywords:** Viral inactivation, MS-2 bacteriophage, Capsid protein, Hydraulic shock, Shockwaves, Hydrodynamic cavitation

## Abstract

This study investigates the effect of hydraulic shock waves on inactivation of MS-2 bacteriophage, a norovirus surrogate. A falling circular jet of water spiked with the MS-2 (∼1000 PFU/mL) was repeatedly impacted by a rotating blade, resulting in occurrence of hydraulic shock waves within the liquid region adjacent to the impact. The proof-of-concept rotational generator of hydraulic shock treating 9 L of water spiked with viruses was able to achieve 3 logs reduction of viral plaque count within 80–100 liquid passes at moderate blade impact velocities (namely, 70 and 88 m/s) despite the water temperature not exceeding 40 °C and no detectible cavitation. Within the first 20 liquid passes, most MS-2 capsid proteins were degraded, with their concentration reduced from 22 μg/mL to only 7.3 μg/mL. Due to the lack of further capsid protein destruction, additional reduction in MS-2 plaque count in subsequent 80 passes is indicative of damage inflicted to the viral recognition receptors. All this suggests that shockwaves of moderate amplitude (few tens of MPa) alone are sufficient for effective viral inactivation. Considering this and the device's good scalability potential, rotational hydraulic shock generators could prove effective in treating virus-contaminated waters.

## Highlights

• Waterborne MS-2 bacteriophage was inactivated by impact-induced hydraulic shock.
• Shockwaves of few 10s of MPa were generated by rotational blade impacting water jet.
• Nearly 3 log reduction of viral infectivity after 80–100 liquid passes.
• Inactivation mechanism in initial stage by viral capsid protein destruction.
• Inactivation after initial stage likely by damage to viral recognition receptors.

## Graphical abstract

**Figure:**
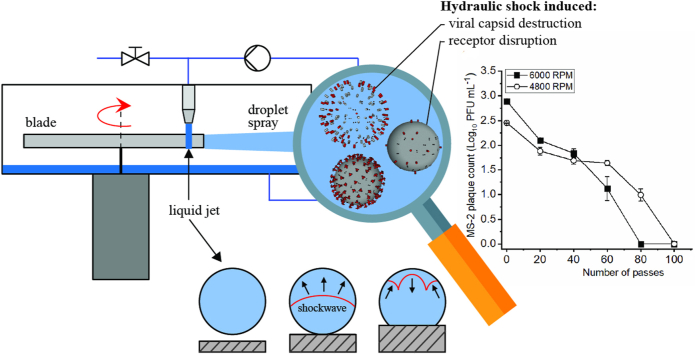

## 1 Introduction

In the light of climate change and continually growing global population, maintaining safe drinking water is becoming increasingly challenging. Water contamination with enteric viruses (noroviruses, sapoviruses, rotaviruses, enteric adenoviruses, and astroviruses) poses a significant risk to public health. These viruses can persist for extended periods in water and can still cause infections even when highly diluted [1]. To prevent epidemic outbreaks, disinfection is key in water treatment as it removes pathogens. Several different methods for inactivation of waterborne viruses have been devised, including chemical methods (e.g. chlorination, ozonation), physical methods (e.g. membrane filtration, ultraviolet radiation) and other methods such as photocatalytic disinfection, electrochemical disinfection and cavitation [2].

Among the variety of water disinfection methods, hydrodynamic cavitation (HC) is one of the most promising emerging technologies, requiring no added chemicals for operation. The most common types of HC-generating machinery include orifices, Venturi constrictions, vortex diodes and rotor-stator interaction-based rotational devices [3]. Possible mechanisms of viral inactivation in HC-based water treatment devices include local surges in pressure and temperature after the collapse of cavitation vapor structures, as well as creation of reactive chemical species in the process [4,5]. Due to the complexity of cavitation-related phenomena, the contribution of individual mechanisms towards viral inactivation remains unclear. Nevertheless, several studies have demonstrated that shock waves alone have the capability to inactivate microorganisms. Fujiwara et al. [6] investigated the effect of impulsive load generated by exploding wire and an explosive on the cell rupture of various microorganisms, noting an inverse correlation between organism size and lethal shock wave amplitude. Leighs et al. [7] utilized flyer-plate impact to induce high pressure peaks on the order of several GPa in confined liquid contaminated with *E. coli*, resulting in destruction of over 99 % bacteria after a single impact. Nevertheless, even shock waves of much lower amplitudes have been demonstrated effective in water disinfection provided that sufficient amount of energy is delivered per volume unit of liquid. Vilkov et al. [8,9]employed a vessel with electric discharge to disinfect water, showing up to 8-log reduction in concentration of *E. coli* and MS-2 coliphage after delivering approximately 2 J/mL of energy to the liquid. Comparable order of effectiveness was achieved by Rutberg et al. on *E. coli* [10] using a similar electric discharge apparatus.

Although a highly effective means of water disinfection, the methods for shockwave generation presented so far have major drawbacks due to their limited scalability (electric discharge) or inability to facilitate continuous use (explosions in confined liquid). To assess the feasibility of shock wave disinfection for large water throughput, a different mechanism of shock wave generation is required. A promising route to attain robust and scalable shock wave generation is by high-speed impact a rotating surface onto a body of liquid. For many decades, rotary devices have been used in simulators of rain erosion [11,12] and cavitation erosion [13], implementing a whirling arm to impact liquid droplets or jets at high velocity often exceeding 100 m/s. Erosion driven by high-speed droplet impacts is highly problematic in steam turbines, wind turbines and aircraft [12,14] and can be up to three orders of magnitude faster than hydrodynamic cavitation erosion of the same material used in pumps or turbines [13]. This raises a legitimate question whether impact-generated hydraulic shocks could further improve viral inactivation effectiveness compared to HC that has itself already been successfully employed in inactivation of different viruses, including MS-2 [15], potato Y [16] and phi-6 [17].

**Fig. 1:**
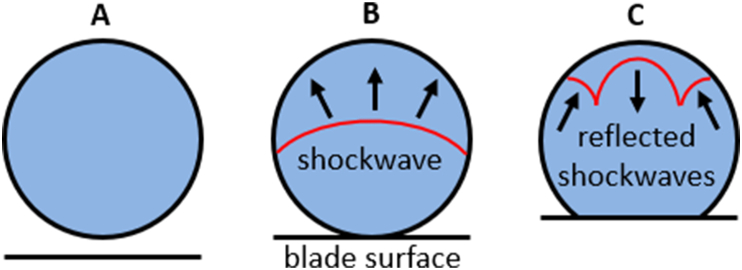
Idealized schematic representation of shockwave generation by impact of blade surface against the water jet (A – unperturbed jet before impact; B – formation of a compression shockwave after impact; C – formation of rarefaction shockwaves after compression shockwave reflection). Arrows mark the shockwave propagation direction.

Unlike HC where shock waves and other viral inactivation mechanisms are a result of vapor structure collapses, high-speed impacts of the liquid against the solid surface immediately result in formation of hydraulic shock waves due to the liquid compression and deformation in contact with solid [18,19], as schematically depicted in Fig. 1. Shock waves of positive amplitude travel the liquid jet or droplet (Fig. 1B) until reaching the liquid surface on the side opposite to the point of impact. Then, the shock waves are reflected towards the focal point of the jet or droplet (Fig. 1C), producing a region of negative pressure that can manifest as cavitation (i.e., formation and collapse of vapor bubble within the liquid droplet or jet) when impact velocity exceeds approximately 100 m/s [20]. Therefore, high-speed liquid-solid impacts can also include cavitation, not as the main driving mechanism of shock wave generation but as an intermediate process producing additional shock waves, potentially enhancing the viral inactivation effect of the initial hydraulic shock.

In the present study, hydraulic shocks were generated by a plate-shaped rotor repeatedly impacting a jet of MS-2 (a norovirus surrogate) virus-spiked water, with the aim of investigating the effectiveness of such a setup for viral inactivation. As mentioned, the MS-2 is a widely used surrogate for waterborne viruses. This F-specific RNA coliphage is small, nonenveloped and spherical, which means that it is generally resistant to chemical disinfectants and environmental factors such as temperature changes, desiccation, and osmotic pressure. Because of its excellent durability, it is routinely used as a quantitative marker and a fecal bioindicator [21,22] for the effectiveness of antiviral and antiseptic agents, and the efficiency of water treatment plants and filtration devices [[23], [24], [25]].

## 2 Materials and methods

### 2.1 Hydraulic shock generator

**Fig. 2:**
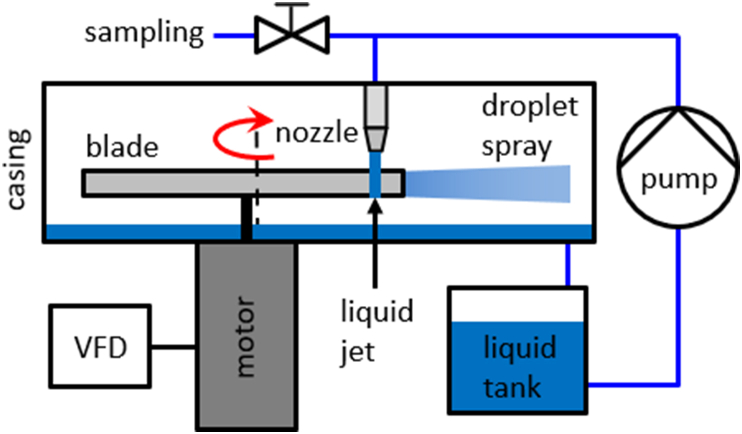
Scheme of the RGHS-based system used to process virus-spiked water.

**Fig. 3:**
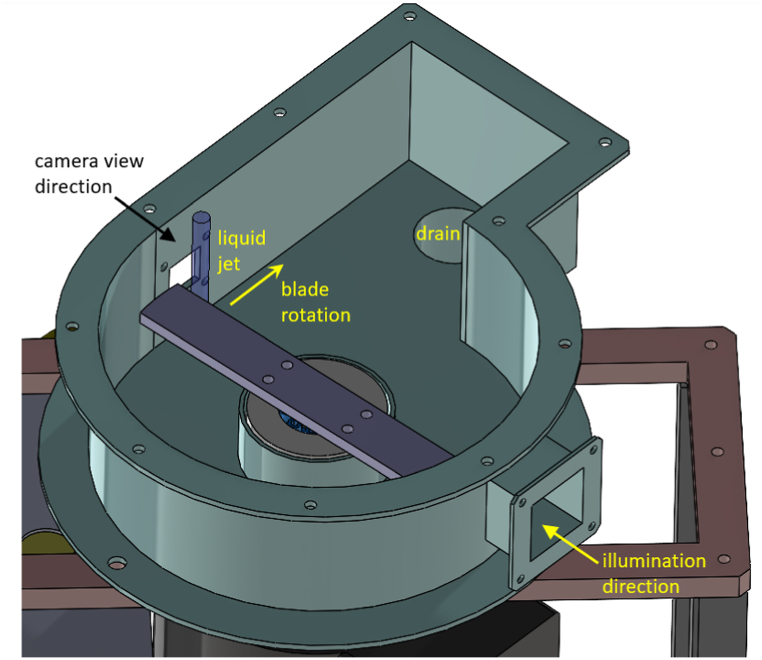
3D model of the rotational generator of hydraulic shock and its reactor vessel (cover not shown).

Viral inactivation experiments were performed on a rotational generator of hydraulic shock (RGHS) as depicted in Fig. 2, Fig. 3. The RGHS comprised a water tank, a circulating pump, and a reactor vessel with a rotating blade. 9 L of water spiked with a MS-2 bacteriophage at approximately 10^3^ PFU/mL was introduced to the RGHS tank and pumped by a gear pump at a flow rate of 18 L/min into the reactor vessel where the liquid exited vertically through a 12 mm nozzle, forming a falling jet with resulting exit velocity of 2.65 m/s. The jet was impacted by a symmetric flat blade of 10 mm height twice per blade revolution, with its rotational speed *f* controlled by a variable frequency drive (VFD) that supplied power to the electric motor. Upon each impact, the falling jet vigorously disintegrated into a spray of droplets that was contained by reactor vessel casing walls and then drained back into the tank. To investigate hydrodynamic effects of the RGHS blade impacting the water jet, the impact process was recorded by a high-speed camera (Photron Fastcam Mini UX100) at 40,000–200,000 frames/second and 4 μs exposure time.

At both blade rotational speeds used (namely, *f* = 4800 RPM and *f* = 6000 RPM, corresponding to 70 m/s and 88 m/s impact velocity, respectively), samples for viral infectivity assay were taken at 0, 20, 40, 60, 80 and 100 liquid passes (duration of one pass was 30 s). Throughout the experiments, the water temperature was maintained below 40 °C by immersing ice-based cooling packs into the tank and replacing them as they melted. This way, it was ensured that despite the power dissipation of over 1 kW in the reactor vessel, the water temperature did not contribute towards inactivation of the virus.

### 2.2 Virus propagation and infectivity assay

Protocols for propagation and infectivity of MS-2 bacteriophage were in accordance with the standard method of the International Organization for Standardization [22]. The host bacterial strain *E. coli* CB390 was maintained at 37 °C on TYGA solid media (15 g L^−1^ of Difco agar; 10 g L^−1^ of tryptone (Difco), 1 g L^−1^ of yeast extract (Difco), 8 g L^−1^ of NaCl, 100 mg/L of ampicillin (Sigma) and 1.93 g L^−1^ of MgCl2^.^6H20). Its overnight culture was prepared in a 15 mL glass tube containing 4 mL of TYGB medium (TYGA without the agar) and was incubated at 37 °C and 250 rpm (aerobic incubation). Then, 160 μL of the overnight culture was inoculated into fresh TYGB medium and after 2 more hours of aerobic incubation the log phase host culture was ready to use. The MS-2 stock was prepared in three propagation cycles. For each cycle, 200 μL of filtered phage suspension (0.22 μm filter, Millipore Corp.) was inoculated into 4 mL of log phase host culture. After an overnight incubation (37 °C and 250 rpm), 1 mL of suspension was centrifuged at 4000×*g* for 20 min and the supernatant was passed through a 0.22 μm Millipore filter. For the next cycle, 200 μL of this filtrate was further inoculated into a fresh log phase host culture. The final filtered stock contained ∼13 log_10_ PFU mL^−1^. To prepare the working bacteriophage titer suspension, the stock was firstly serially diluted in SM buffer (5.8 g L^−1^ of NaCl, 2 g L^−1^ of MgSO4∗7H2O, 0.01 % of gelatin and 50 mM Tris base; pH7.5 [26,27]) and finally diluted in tap water to a concentration of ∼2 log_10_ PFU mL^−1^. A low viral titer was selected, because concentrations of viruses like MS-2 obtained from real-world wastewater samples (1–4 log_10_ PFU mL^−1^) and sewage-impacted wetlands (∼1.5 log_10_ PFU mL^−1^) have been found to be relatively low [28,29]. Before the hydraulic shock experiment, 9 L of tap water spiked with 1 mL of ∼1∗10^6^ PFU/mL of MS-2 in SM buffer contained a minute concentration of organic load (organics introduced from 1 mL of inoculum with the virus and buffer): ∼2 log_10_ PFU mL^−1^ of MS-2 harboured ∼3 pg/mL of total capsid protein (estimated from Micro BCA™ measurements-see section 2.4); ∼0.6 mg/L of NaCl; ∼0.2 mg/L of MgSO4∗7H2O; ∼0.01 mg/L of gelatin; and ∼5 μM Tris base. Moreover, according to the water quality analysis determined by the National laboratory of health, environment and food (NLZOH; https://www.ljubljana.si/sl/moja-ljubljana/varstvo-okolja/stanje-okolja/kakovost-vode-aktualni-podatki/) tap water in Ljubljana normally contains the next impurities: ammonium: 3 μg/L; fluoride: 0.1 mg/L; hydrogen carbonates: 260 mg/L; calcium: 52 mg/L; potassium: 0.3 mg/L; oxygen: 9.5 mg/L; chloride: 2.7 mg/L; magnesium: 28 mg/L sodium: 0.7 mg/L; nitrates: 7.5 mg/L; NO_3_ orthophosphate: 6 μg/L and sulfate: 3.7 mg/L; 88 % oxygen saturation; and pH value of 7.6.

Until the beginning of experiments, within the rotational generator of hydraulic shock, viral titers were stored on ice in an expanded polystyrene box. For sampling, before and after each operating interval (within the tested device), 10 mL of suspension was collected using a pipette and 9 mL of it was mixed with 3 mL of 4 × SM buffer and stored at 4 °C for 5 days. Before each sampling, a small volume of liquid was drained from the sampling valve to remove any dead volume of liquid remaining unprocessed and uncirculated in the piping. For virus quantification, a double-layer plaque assay was employed. For each technical repetition 5 mL of melted ssTYGA medium (TYGA with 7 g L^−1^ of Difco agar) was prepared in a 15 mL glass tube and was placed into a water bath at 55 °C. Then 100 μL of log phase host culture and 1 mL of premixed sample (or its dilution) were added into the tube (for the blank sample 1 mL of SM buffer was used instead). The tube was then covered, briefly shaken and the mixture was poured onto a TYGA petri plate. After an overnight incubation at 37 °C, the number of plaques was counted, and their concentration was calculated by considering the dilution factor and plating volume (PFU/mL). For each sample, three technical repetitions were prepared. Reported values are the mean value of three independent biological treatments, and the error bars represent standard deviations [30].

For above experiments, MS-2 bacteriophage removal was determined by calculating the specific decay rate constant (*μ*) – Eq. (1):

(1) $$\mu =\ln\left(\frac{\ln\;X_{0}-\ln\;X_{f}}{{\text{No}}_{total\;passes\;}}\right)$$

Specific decay rate is the slope of the MS-2 decay curve, and a higher specific decay rate value corresponds to higher MS-2 destruction efficiencies. *X*_*0*_ is plaque count per milliliter at the beginning of treatment; *X*_*f*_ is plaque count per milliliter at the end of treatment and *No*_*total passes*_ is the number of full volume liquid passes at the end of the experiment.

### 2.3 Operational controls

To ensure that the tested devices were free of microorganisms, a cleaning protocol was implemented before and after each experimental run. This consisted of one rinse with tap water (running the rotational generator of hydraulic shock filled with tap water for 1 min), one 5 min long rinse with 70 % ethanol, and finally six successive device volume rinses with tap water (each lasting 1 min). All of the drained/rinsed water was disposed after an overnight exposure to active chlorine tablets. To determine the effectiveness of washing between treatment experiments, the tap water from the last rinse was sampled and quantified by plaque counts. Additionally, before each experimental run, the effect of possible MS-2 attachment on the interior surfaces of devices was tested. For this purpose, samples were taken immediately before and after filling the devices with a viral working suspension, and the plaque counts were compared between both.

During the whole course of operation, the temperature of the MS-2 spiked water sample inside the RGHS, was monitored using a PT100 A type resistance thermometer (uncertainty of ±0.2 °C) so that water temperature at any time did not exceed 40 °C.

Apart from the rotating blade, the circulating pump could also have an effect on the MS-2 infectivity. Therefore, the sole effect of pumping the sample through the circulating pump of the rotational generator of hydraulic shock on MS-2 bacteriophage infectivity was assessed. For this purpose, 20 sample passes of virus-spiked tap water (∼3 log_10_ PFU mL^−1^ of MS-2) were made with the rotating blade turned off (no hydraulic shock imposed) and the circulating pump pumping at 2 sample passes per minute.

### 2.4 Hydraulic shock effect on capsid protein destruction of MS-2 virus

The effect of hydraulic shock generated by blade impacts at 6000 RPM on the destruction of capsid proteins of the MS-2 virus was assessed using the Thermo Scientific™ Micro BCA™ Protein Assay Kit. To ensure a sufficiently high concentration of capsid proteins for detection using this assay (in range of 2–40 μg/mL), a high initial MS-2 spiking concentration of 3.4∙10^9^ pfu mL^−1^ was utilized at the onset of the hydraulic shock treatment. The assay uses bicinchoninic acid (BCA) as the detection reagent for Cu^+1^, which is formed when Cu^+2^ is reduced by protein in an alkaline environment [31]. A purple-colored reaction product is formed by the chelation of two molecules of BCA with one cuprous ion (Cu^+1^). This water-soluble complex exhibits a strong absorbance at OD_562_ that is linear with increasing protein concentrations. The macromolecular structure of protein, the number of peptide bonds and the presence of four amino acids (cysteine, cystine, tryptophan and tyrosine) are reported to be responsible for color formation with BCA [32]. Firstly, the working reagent is prepared by mixing 75 μL of MA, 72 μL of MB and 3 μl of MC. Then, 150 μL of sample is pipetted into a PS microplate well (for background subtraction 150 μl of MilliQ water was used) and immediately after that 150 μL of the working reagent is added. Microplate is mixed thoroughly on a plate shaker for 30 s, sealed using parafilm and incubated at 37 °C for 2 h. After incubation, the adsorbance (562 nm) was measured. From OD_562nm_ scans, capsid protein concentration was calculated using the calf serum albumin protein standard (CSA; SI-N4637 Sigma) calibration curve, which was constructed from OD_562nm_ measurements of serially diluted CSA standard.

## 3 Results and discussion

### 3.1 Hydrodynamic characteristics of the RGHS

**Fig. 4:**
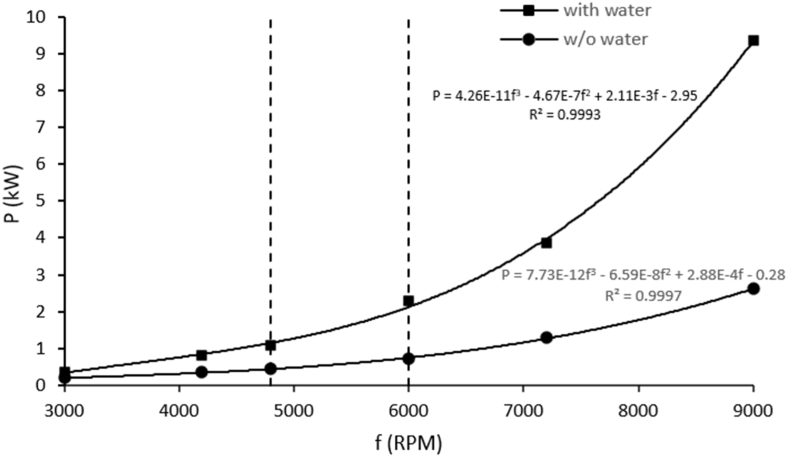
Power consumed by the rotational generator of hydraulic shock with and without impacting the water jet. Data points are fitted by third order polynomials.

The only RGHS operating parameter varied in the present study was the rotational speed of the blade. As the blade impacts the falling water jet, a part of its kinetic energy is transferred to the liquid during impacts, whereas the remaining energy is dissipated through the drag force of the reactor vessel air acting upon the blade. The forces of overcoming drag and accelerating the liquid can be expected to increase approximately in proportion to the square of the impact velocity. This implies a cubic relation between the device's power consumption *P* and the blade's rotational speed *f*, since the power demand of overcoming aerodynamic drag and accelerating the liquid equals the product of velocity and corresponding forces integrated over the radial extent of the blade. Not surprisingly, Fig. 4 reveals that the power drawn by the rotational generator of hydraulic shock increases nonlinearly with blade rotational speed, rising from 0.4 kW at 3000 RPM to 9.4 kW at 9000 RPM (note a very good fit of the third order polynomial *P*(*f*) in Fig. 4 for operation with and without water, confirming the cubic relation between *P* and *f*). To avoid excessive power consumption and resulting rapid heating of the virus-spiked water in the system, 4800 RPM and 6000 RPM blade rotational speeds were selected for viral inactivation experiments (power consumption of 1.1 kW and 2.3 kW, respectively). Under these conditions, approximately 70 % of provided energy was consumed for the impaction and acceleration of the water jet.

### 3.2 Jet impact phenomenon

**Fig. 5:**
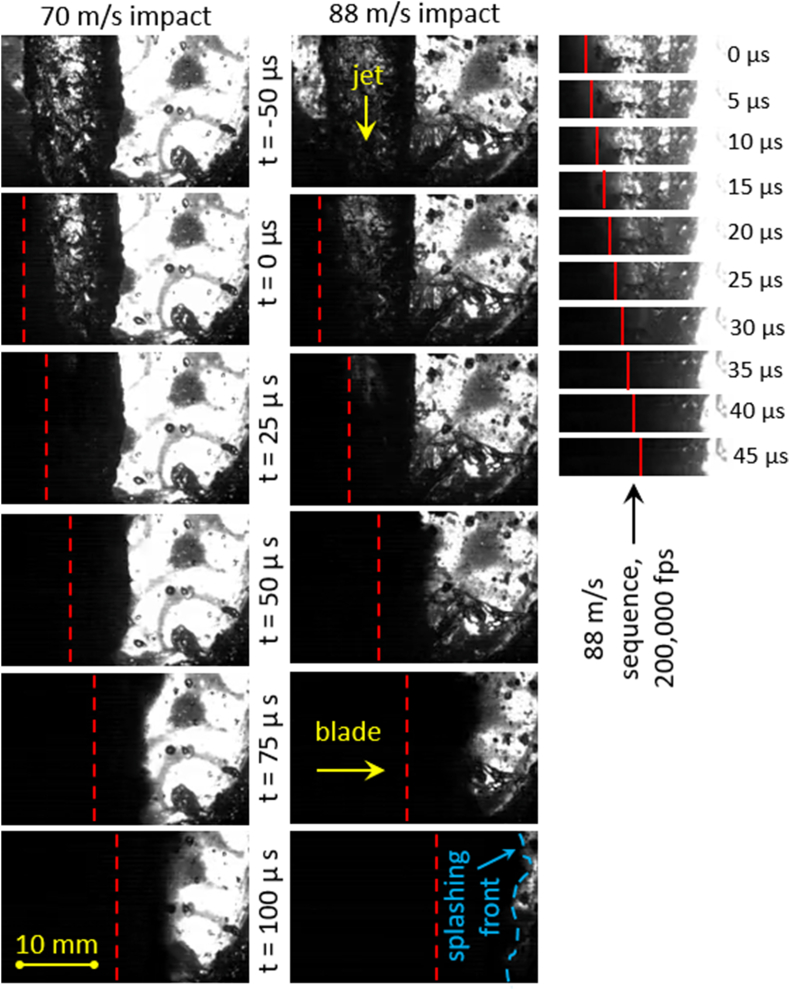
High-speed image sequences of rotating blade impacting the water jet at 70 m/s and 88 m/s, 4 μs exposure time in all images.

The effect of rotating blade impact on the falling water jet can be observed in a succession of high-speed images presented in Fig. 5 for a fully developed jet impacted at 70 m/s (40,000 frames/second) and 88 m/s (40,000 and 200,000 frames/second). As the blade contacts the jet surface (*t* = 0 μs), the liquid on the side of the impact is compressed while the opposite-side boundary of the jet initially does not move. Shockwave formation is likely at such impact velocities [20], but difficult to observe with a high speed camera [19] unless Schlieren imaging or a similar technique is used. In our case, although shockwaves were not visible, their presence is indicated by a vigorous breakup of the liquid jet after impact. Within 25 μs from the time of impact the liquid becomes opaque as it disintegrates into a curtain of fine droplets (similar behavior was also observed by Keil et al. [13] for 70 m/s impact velocity). After t ≈ 50 μs, the water jet is fully disintegrated, forming a rapidly accelerating front of splashing liquid that eventually obstructs the viewing area. It is worth noting that relatively high concentration of slow airborne droplets was observed in the reactor vessel (also visible in Fig. 5 to the right of the jet), indicating the likelihood of secondary impacts of droplets with the blade, thus potentially enhancing the viral inactivation effect.

For a more detailed analysis of disturbance propagation in the jet, additional high speed videos were recorded at 200,000 frames/second (successive images of an 88 m/s impact shown in the right column of Fig. 5). From these image sequences, we can estimate that the droplet spray accelerates to 95 m/s and 140 m/s for the impact velocity of 70 m/s and 88 m/s, respectively. The fact that the droplet spray velocity significantly exceeds the blade velocity is a strong indication of shockwave presence. On the other hand, since both impact velocities are below 100 m/s, the extent of cavitation induced by shockwave reflection and focusing will likely be minimal [20], particularly when considering the nonuniform and visibly perturbed surface of the water jet in the present study further reducing the intensity of shockwave focusing (note that studies conducted so far considered an ideally round and smooth jet). Therefore, we expect the viral inactivation mechanisms to be largely attributable to the exposure of virus to initial and reflected shockwaves, with effects potentially generated by cavitation (e.g., local temperature surges and reactive chemical species formation) likely insignificant. Considering nonlinearly increasing power requirements, the feasibility of operating the rotational generator of hydraulic shock at very high impact velocity to enhance viral inactivation through generating strong cavitation is questionable.

Further insight into the mechanisms of viral inactivation by shockwaves is provided by Vilkov et al. [8,9]. According to the authors, exposure to a single shockwave can result in severe mechanical damage of microorganisms through the mechanism of Rayleygh-Taylor instability if the shockwave front thickness is less than the size of protective structures such as cell walls or viral capsids [8]. According to the authors, a shockwave amplitude between 60 and 135 MPa is required for microbe destruction by a single shockwave, depending on microorganism structure and type. In contrast, Grünbein et al. [33] estimated a shockwave damage threshold of 40 MPa by crystallography of protein crystals damaged by an x-ray free-electron laser. In our setup, a small portion of liquid (say, around 1 % for our range of impact velocity) close to the initial contact point of blade impact can be subjected to pressure close to the theoretical water hammer pressure *ρvc* [20] (*ρ* = 1000 kg/m^3^ is water density, *c* = 1500 m/s is the speed of sound in water, *v* is impact velocity), which equals to 105 MPa and 132 MPa at 70 m/s and 88 m/s impact velocity. This indicates that the pressure amplitude in the immediate impact area may suffice for single-pass viral inactivation. However, the remaining part of the liquid can be expected to experience substantially lower peak pressure [20], implying that several liquid passes would be required for full viral inactivation, as was also the case in a study utilizing HC for MS-2 inactivation [15]. The viral inactivation contribution of the high pressure region near the impact point relatively to the larger lower pressure liquid zone is unclear and requires further investigation.

It is worth noting that true water hammer pressure conditions can only apply up to a point when a critical contact angle is reached (the angle increases with the impact velocity, and so does the volume fraction of liquid exposed to pressure amplitudes close to theoretical water hammer pressure). Liquid column compression beyond this point results in cessation of shockwave formation, with existing shock wave detaching and diminishing in amplitude as it travels across the liquid [20]. Considering this, a more realistic peak shockwave pressure amplitude *p*_max_ within most of the liquid domain can be estimated using a correction factor *k* that represents the ratio of *p*_MAX_ to theoretical water hammer pressure *ρvc*. Employing pressure distribution results presented in Ref. [20], an estimate of *k* ≈ 0.27 can be made for both impact velocities used in the present study. Using the peak shockwave pressure equation, we obtain *p*_max_ = 28 MPa at *v* = 70 m/s and *p*_max_ = 36 MPa at *v* = 88 m/s. Nevertheless, the estimated *p*_max_ value is still by an order of magnitude higher than the typical peak pressure in a Venturi tube where Petkovšek et al. [34] optically estimated *p*_max_ ≈ 2 MPa in the cavitation bubble collapse zone for a fully developed HC regime. Considering the much higher RGHS shockwave pressure compared to a typical HC Venturi setup, and the fact that the main mechanisms of chemical oxygen demand (COD) and biochemical oxygen demand (BOD) reduction in HC are pressure surges [35,36], an RGHS installed within a water cleaning station could be employed not only for the inactivation of microorganisms, but also for the degradation of chemical and organic load.

### 3.3 Operational controls

The employed washing protocol successfully removed all bacteriophages between different experimental runs. Thus, no plaques were observed on any of the cleaning control plates. Additionally, we found that plaque counts of samples that were taken immediately before and after the filling of the tested devices with the viral suspension, differed only slightly (data not shown). Therefore, viral attachment to the inner surfaces of the tested devices was not a significant issue.

The experiments performed with the RGHS blade stationary and pump circulating water at 2 sample passes/min showed that the virus infectivity was reduced by only 0.01 log10 PFU mL-1 after 20 passes (from initial 2.45 log10 PFU mL-1) of a 9 L sample, thereby eliminating the pumping itself as a mechanism of virus inactivation. These samples (blade turned off with functional pump), after the pumping experiment and prior to virus quantification, were stored at 4 °C for 5 days, exposing viruses to impurities, which are normally present in tap water, for a period of 5 days. This proves that in comparison to virus inoculum prepared in SM buffer (optimal buffer for virus survivability prepared in MilliQ water) the tap water impurities and no effect on virus infectivity.

**Fig. 6:**
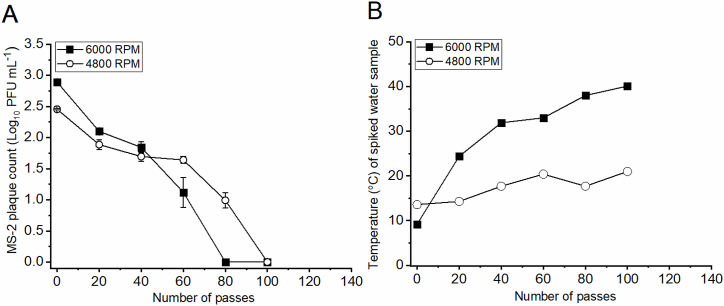
The effect of hydraulic shock generated by rotational generator of hydraulic shock at 4800 RPM or 6000 RPM blade rotational speed on the infectivity of MS-2 bacteriophage (Frame A), with corresponding temperature of the MS-2 spiked water in the liquid tank (Frame B).

**Fig. 7:**
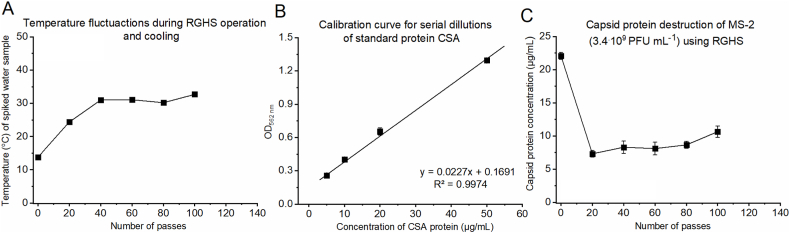
The effect of treatment rotational generator of hydraulic shock at 6000 RPM, on the capsid protein destruction of MS-2 virus (Frame C) and corresponding temperature (Frame A); CSA calibration curve constructed from OD_562nm_ measurements of serially diluted CSA standard (Frame B).

The temperature of the sample (Fig. 6B) was monitored before, during and after the spiked sample treatments within the rotational generator of hydraulic shock. When the rotating blade of the RGHS was spun at *f* = 6000 RPM, the pre-treatment temperatures were ∼10 °C and the post-treatment temperatures ranged from 39 °C (Figs. 6B and 6000 RPM) to 32 °C (Fig. 7A). During the experiment, the expired cooling packs within the water tank were substituted with freshly frozen ones to maintain the cooling effect. Lower temperatures were measured at *f* = 4800 RPM, reaching only ∼21 °C towards the end of the experiment (Figs. 6B and 4800 RPM). Therefore, during all treatment runs, the temperatures of the MS-2 spiked water samples stayed below 40 °C. According to Khalil et al. [37] temperatures ranging below 50 °C have no significant effect on the infectivity of the MS-2 bacteriophage even for longer time periods that stretch for up to 12 h, meaning that water temperature can be eliminated as the viral inactivation mechanism.

### 3.4 Influence of hydraulic shock on the infectivity of MS-2 virus

The effect of hydrodynamic shock developed due to rotor impacting the water jet on the infectivity of MS-2 bacteriophage is presented in Fig. 6A (MS-2 infectivity) and Fig. 6B (temperature of spiked water sample). In these experiments, the spiked water samples were treated for 50 min, corresponding to 100 sample passes. At f = 6000 RPM, the initial bacteriophage titer (2.8 log_10_ PFU mL^−1^) was reduced by 0.8 log_10_ PFU mL^−1^ after just 20 sample passes and was completely inactivated (0 PFU mL^−1^) after 80 passes through the reactor vessel of the RGHS. According to these measurements a ∼3 logs reduction was achieved after 40 min of operation. At 4800 RPM, the initial bacteriophage titer (2.5 log_10_ PFU mL^−1^) was reduced by 0.6 log_10_ PFU mL^−1^ after 20 sample passes and was completely inactivated (0 PFU mL^−1^) after 100 passes through the reactor vessel of the RGHS. Therefore, after 50 min of operation no plaques were observed (∼2.5 logs reduction).

Specific decay rates (*μ*, Eq. (4)) of MS-2 virus after hydraulic shock treatments at 6000 RPM and 4800 RPM were calculated from the above data and are presented in Table 1. Higher *μ* values correspond to higher MS-2 destruction efficiencies. The decay rates for the removal of MS-2 (with a starting concentration of ∼5^.^10^2^ PFU/mL) from tap water using the hydraulic shock treatments at 6000 RPM and 4800 RPM were −2.5 and −2.9, respectively. In our previous study we employed hydrodynamic cavitation generated within the blow-through Venturi set-up (BTV) for the treatment of a similar MS-2 starting titer (∼5^.^10^2^ PFU/mL) achieving a relatively low *μ* value of −3.5 [38]. This *μ* value is substantially lower to that of the RGHS at 6000 RPM at a difference of 1.0. BTV experiments were also conducted for the treatment of high titers of bacteriophage phi6 (surrogate for SARS-CoV-2) [39] and potato virus Y (PVY) [40], reaching *μ* values of −4.2 and −3.3, respectively. Both of these values were significantly lower than those observed for MS-2 virus within the RGHS, indicating lower per-pass efficiency of BTV in comparison with the RGHS. The past work with the BTV showed that this device achieved a ∼5 logs reduction on a high MS-2 titer and therefore met the standards set by the US Environmental Protection Agency (EPA) for virus removal in water purifiers (at least a 4-log reduction (99.99 %) needed) [41]. Because the efficiency of MS-2 destruction by hydraulic shock is higher to that of the BTV, its application is even more aligned with the EPA standards.

**Table 1:** Specific decay rate of MS-2 virus after RGHS treatments at 6000 RPM and 4800 RPM in comparison to the Blow-through Venturi set-up treatments performed in previous studies with MS-2 virus (Kosel et al., 2017b, superscript A); with phi6 virus (Zupanc et al., 2023b, superscript B); and with PVY virus (Filipić et al., 2022b, superscript C).

Treatment methodology	Intensity of treatment	Treated virus	Number of passes	*X*_*0*_ (PFU/mL for bacterioph. or copies_RNA_/mL for PVY)	*X*_*f*_ (PFU/mL for bacterioph. or copies_RNA_/mL for PVY)	*Logs reduction*	*μ* value
Blow-through Venturi set-up (hydrodynamic cavitation)	7 bar pressure difference	High titer (2^.^10^8^ PFU/mL) of bacteriophage MS-2	1040 ^***A***^	2.7^.^10^8^^***A***^	1.9^.^10^3^^***A***^	5.1 ^***A***^	−4.5 ^***A***^
		Low titer (5^.^10^2^ PFU/mL) of bacteriophage MS-2	208 ^***A***^	5.9^.^10^2^^***A***^	0 ^***A***^	2.8 ^***A***^	−3.5 ^***A***^
		High titer (4^.^10^6^ PFU/mL) of bacteriophage phi6 (surrogate for an enveloped virus-less stable at high pressures)	1000 ^***B***^	4.4^.^10^6^ ^***B***^	0 ^***B***^	6.6 ^***B***^	−4.2 ^***B***^
		High titer (1^.^10^8^ copies_RNA_/mL) of PVY (potato virus Y)	500 ^***C***^	∼1^.^10^8^ ^***C***^	0 ^6***C***^	∼6 ^***C***^	−3.3 ^***C***^
RGHS (hydraulic shock)	6000 RPM	Low titer (6^.^10^2^ PFU/mL) of MS-2	80	6.3^.^10^2^	0	2.8	−2.5
	4800 RPM	Low titer (3^.^10^2^ PFU/mL) of MS-2	100	3.2^.^10^2^	0	2.5	−2.9

### 3.5 Influence of hydraulic shock on capsid protein destruction of MS-2 virus

Having demonstrated the effectiveness of hydraulic shock waves on viral inactivation, understanding the mechanism thereof is also of great importance. Hydraulic shock has the potential to cause structural damage to various components of a virus, including the viral coat, capsid protein, virus genome (nucleic acid), or the host recognition receptors present on the viral capsid. Since there had been no definite prior knowledge of the mechanism by which hydraulic shock impacts viral infectivity, the effect of shockwaves generated in the RGHS on MS-2 capsid protein destruction was investigated in an additional experiment at *f* = 6000 RPM, using a high initial titer of 3.4 × 10^9^ PFU mL^−1^ (Fig. 7). At the beginning of this experiment the concentration of MS-2 capsid proteins was around 22 μg/mL. However, after the first 20 treatment passes through the reactor vessel of the RGHS, capsid protein concentration rapidly dropped to only 7.3 μg/mL (a reduction of 67 %, Fig. 7C). During the next 80 treatment passes, MS-2 capsid protein concentration remained almost constant (8–10 μg/mL) until the end of the experiment. Hence, the majority of capsid proteins were degraded during the first 20 treatment passes within the RGHS.

Based on results presented in Fig. 7C, it appears that the initial hydraulic pressure shock causes the most intense capsid protein damage, which largely explains the initial 1-log reduction in MS-2 viral infectivity observed during this treatment period (Figs. 6A and 6000 RPM curve). Since no further capsid protein destruction occurred within the subsequent 80 treatment passes, the additional 2-log reduction in MS-2 viral infectivity observed during this period (Figs. 6A and 6000 RPM curve) is attributable to other viral damage mechanisms, most likely the disruption of MS-2 recognition receptors present on the viral capsid [42]. Even slight damage to these recognition receptors can result in the loss of infectivity of the virus, as without functional receptors, the MS-2 bacteriophage cannot bind to its *E. coli* host [43]. Therefore, we have demonstrated that hydraulic shock simultaneously causes damage to the MS-2 virus on two separate levels: (a) capsid protein destruction and (b) other damage mechanisms such as disruption of recognition receptors.

### 3.6 Energy efficiency and scalability considerations

The water treatment energy efficiency of the presented RGHS device can be estimated from the power consumption and required time to achieve the desired viral inactivation effect. At 4800 RPM, RGHS operated at 1.1 kW power for 100 passes (50 min), resulting in specific energy consumption of 102 kWh/m^3^. At 6000 RPM, RGHS operated at 2.3 kW power for 80 passes (40 min) until complete disinfection, resulting in specific energy consumption of 170 kWh/m^3^. Energy consumption per log reduction of viral infectivity was 41.7 kWh/m^3^/order at 4800 RPM and 84.7 kWh/m^3^/order at 6000 RPM. For comparison, water disinfection by boiling from initial temperature of 12.5 °C (as in the 4800 RPM experiment) consumes approximately 102 kWh/m^3^, or 41.7 kWh/m^3^/order. Therefore, the specific energy efficiency for water treatment by presented rotational generator of hydraulic shock is comparable to the boiling process, but by 2–3 orders of magnitude higher than processes such as UV radiation, chlorination or ozonation [2]. Consequently, the potential utility of presented device is in preparation of drinking water where the use of chemicals is unfeasible or undesired, and relatively high energy consumption comparable to the boiling process is acceptable.

Having said that, significant reduction in specific energy consumption can be expected if the following measures are implemented: a) optimization of blade rotational speed to achieve minimum specific energy consumption; b) utilization of waste heat (i.e., operating without cooling packs and allowing the water temperature to rise above the thermal inactivation temperature); c) optimization of impact surface geometry; d) upscaling to increase the fraction of energy consumed for impacting the liquid. Due to its design, the scaling of the RGHS device for higher processing capacity is relatively straightforward, employing one or more of the following measures: a) increasing the diameter (and hence, flow rate) of the water jet; b) increasing the thickness of the blade (i.e., larger volume of liquid processed in each impact); c) increasing the number of liquid jets (i.e. arranging several jets in a circular pattern about the blade axis).

## 4 Conclusions

In this paper, the effectiveness of a proof-of-concept rotational generator of hydraulic shock for inactivation of MS-2 bacteriophage virus was investigated. Two different blade rotational speeds were used, resulting in blade-jet impact velocity of 70 m/s and 88 m/s, respectively. Based on measurements of RGHS hydraulic characteristics, MS-2 plaque counts and MS-2 capsid proteins, the following conclusions can be drawn:

1. Moderate impact velocity (70–88 m/s) and hydraulic shock pressure amplitude (28–36 MPa) induced by the impact of the blade on the jet are sufficient to completely inactivate the waterborne MS-2 virus after 80–100 liquid passes. Future research should determine the lower threshold of impact velocity and shockwave pressure amplitudes for effective viral inactivation.
2. Viral infectivity is reduced by a dual mechanism including destruction of capsid proteins in the initial stage of processing, followed by other damage mechanisms such as disruption of the viral recognition receptors.
3. The effect of cavitation on viral inactivation is likely negligible and much higher velocity would be needed to produce substantial HC (not economically feasible).
4. Compared to blow-through Venturi setups, the rotational generator of hydraulic shock can achieve the same degree of viral inactivation in a much lower number of liquid passes (and hence, in less time).
5. High heat dissipation in RGHS suggests that further research should focus on study and optimization of the device's energy efficiency, namely operating at even lower blade rotational speeds and with modified blade geometry should be investigated.
6. The design of the device enables good and straightforward scalability.

Having successfully demonstrated the viral inactivation effectiveness of presented rotational generator of hydraulic shock, one can envision a broad field of applications in preparation of drinking and process water from virally contaminated sources, particularly when chlorination or high temperature treatment is undesirable. Since viruses are only one class of pathogens, further research will benefit from also exploring the antibacterial performance of the device to assess the all-round feasibility of the RGHS for water disinfection.

## CRediT authorship contribution statement

**Benjamin Bizjan:** Writing – original draft, Investigation, Conceptualization. **Gašper Rak:** Supervision, Funding acquisition. **Sabina Kolbl Repinc:** Supervision, Project administration. **Polonca Ropret:** Resources, Methodology. **Janez Kosel:** Writing – review & editing, Writing – original draft, Methodology, Formal analysis.

## Data availability statement

Data available on request from the corresponding author.

## Funding

The authors acknowledge the financial support from the 10.13039/501100004329Slovenian Research Agency (ARIS) research core funding P2-0401, P2-0180 and BI-RS/20-21-013, as well as grant J7-3147.

## Declaration of competing interest

The authors declare that they have no known competing financial interests or personal relationships that could have appeared to influence the work reported in this paper.
